# Mammalian Introns: When the Junk Generates Molecular Diversity

**DOI:** 10.3390/ijms16034429

**Published:** 2015-02-20

**Authors:** Florent Hubé, Claire Francastel

**Affiliations:** CNRS UMR7216, Epigenetics and Cell Fate, Université Paris Diderot, Sorbonne Paris Cité, UMR7216 Epigénétique et Destin Cellulaire, Bâtiment Lamarck B, Case Courrier 7042, 35 rue Hélène Brion, 75013 Paris, France

**Keywords:** intron, splicing, genome, gene, non-coding RNA, transcription, data mining

## Abstract

Introns represent almost half of the human genome, yet their vast majority is eliminated from eukaryotic transcripts through RNA splicing. Nevertheless, they feature key elements and functions that deserve further interest. At the level of DNA, introns are genomic segments that can shelter independent transcription units for coding and non-coding RNAs which transcription may interfere with that of the host gene, and regulatory elements that can influence gene expression and splicing itself. From the RNA perspective, some introns can be subjected to alternative splicing. Intron retention appear to provide some plasticity to the nature of the protein produced, its distribution in a given cell type and timing of its translation. Intron retention may also serve as a switch to produce coding or non-coding RNAs from the same transcription unit. Conversely, splicing of introns has been directly implicated in the production of small regulatory RNAs. Hence, splicing of introns also appears to provide plasticity to the type of RNA produced from a genetic locus (coding, non-coding, short or long). We addressed these aspects to add to our understanding of mechanisms that control the fate of introns and could be instrumental in regulating genomic output and hence cell fate.

*In eukaryotes, the process of making a messenger RNA (mRNA) involves the co-transcriptional excision of introns in the nucleus, whereas joined-exons are exported to the cytoplasm to be translated. As a consequence, introns are inherently non-protein-coding sequences in that they are transcribed but not translated (usually) into protein. Because, and by definition, introns always lie between 2 exons, they were called “Intervening” or INtrons. By opposition, exons are those sequences that are EXpressed and EXported to the cytoplasm.*

## 1. Introduction

In 1993, the Nobel Prize was awarded to Phillip A. Sharp and Richard J. Roberts for their discovery that genes can be split into segments and, as a consequence, transcripts that originate from them are matured into messenger RNA (mRNA) smaller in size. Hence, introns are segments of a gene between exons, which are transcribed but do not participate in the production of the final protein product as they are removed before translation, allowing joining of exons through a process known as splicing. With the discovery that various combinations of segments may be included in the final RNA molecule, this has changed our view on how genetic information is expressed and provided, *a posteriori*, an explanation on how eukaryotes diversify their proteome from just a few genes. We now know that the fate of introns is not simply a matter of being eliminated to allow formation of mRNA coding for proteins.

Introns are classified in four groups based on splicing mechanisms. (1) Spliceosomal introns are found in coding genes of eukaryotes and utilize spliceosomes (large protein-RNA complexes) for splicing [1]. These introns share consensus sequences [2] that include the 5' donor site (MAG|GTRAGT where M is A/C and R is A/G), the branch point sequence covalently linked to the 5' end of the intron and formation of a lariat during the splicing process of this type of intron (TCCTRAY where R is A/G and Y is T/C, A being the branch point nucleotide), the polypyrimidine tract (CnTn) and the 3' acceptor site (MAG|G); (2) Transfer RNA (tRNA) introns are removed by specialized enzymes with endoribonuclease and ligase activities [3]; (3) Group I and (4) Group II introns catalyze their own splicing without the aid of any protein, and where thus named ribozymes [4]. They are found in bacteria, plants and lower but not higher eukaryotes.

Splicing of both Group II and pre-mRNA introns involve the formation of a lariat suggesting that they may be evolutionary related. However, origin and evolution of spliceosomal introns is still a matter of debate, opposing “introns early” to “introns late” theories [5], the main dilemma being on the possible roles of introns in the evolution of eukaryotes.

Being eliminated to allow formation of messenger RNA and inherently non-coding, introns have long been kept in the now famous “junk DNA” drawer. However, and maybe against all odds, high sequence conservation among homologous introns of closely related species suggests functional constraints on intronic sequences throughout evolution [6]. Many studies have now added to the weight of evidence showing that introns can serve a considerable range of biological functions.

We review here aspects of introns that are perhaps less known but made them see a renewed interest lately.

## 2. Introns Account for Half of the Genome—From a Single Nucleotide to One Megabase

Most eukaryotic genes are interrupted by stretches of non-coding segments, *i.e.*, introns, which cover more than 1.7 billion bases pairs and represent 45% to 55% of the human genome (GCRh38, Table 1).

**Figure 1:**
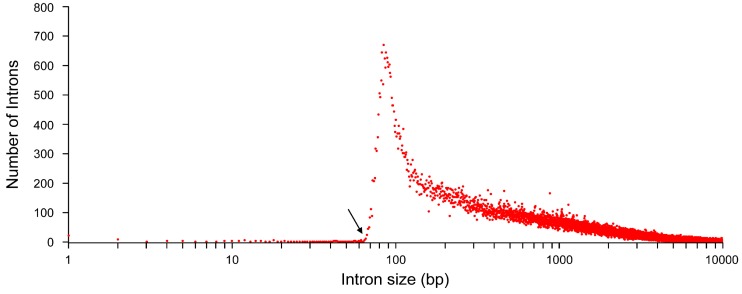
Size distribution of human introns. The human genome data was downloaded from the UCSC main table browser (GRCh38, December 2013 build). Data was processed using tabular software. Analysis was performed using 277,495 introns. The lower size limit for human introns is represented by the dark arrow and is comprised between 60 and 70 bases.

Most introns are above 60–70 base pairs in length as shown in Figure 1. Strikingly, some introns are very small, with about 722 (0.25%) of them being smaller than 73 bases, however, the extremely small introns, especially ≤21 nt, could reflects false introns, or artifactual gaps, generated by automatic alignments. Thus far, the smallest real intron whose splicing was demonstrated is 43 nt in human *ESRP2* gene [7]. In contrast, the longest intron is greater than one megabase (1,043,910 bases) in length, e.g., intron 1 of acid-sensing ion channel 2 (ASIC2; chr17:33,112,068–34,155,977), although this is exceptional since only 35 introns are above 500 kb. Altogether, the average size of introns within coding genes is 5889 ± 19,584 bases (median size of 1520) while introns of non-coding genes are slightly longer, with 7793 ± 23,410 bases (median size of 1707).

Surprisingly, 1394 coding genes (5.33% of total annotated coding sequences, Table 2) and 3303 non-coding genes (38.06% of total annotated non-coding sequences, Table 3) are made of a single exon (single-exon genes; SEG), *i.e.*, they are not interrupted by introns. Although intronless genes are an archetypal feature of prokaryotes, significant amounts of SEG are present in the human genome [8]. Whether SEG are of bacterial origin or came through evolutionary innovations such as retrogenes is still open to discussion [9,10,11]. Interestingly, the high proportion of SEG among non-coding genes would rather be indicative of an evolution from retrotransposons or from ancestral intronless coding genes.

**Table 1:** Distribution of exons and introns across human chromosomes. Human genome dataset was downloaded from the UCSC main table browser (GCRh38, December 2013 build). Data was processed using tabular software. Analysis was performed using the 34,856 genes containing 312,351 exons and 277,495 introns. Caution: introns with the length <30–40 nt likely reflect artifact or error (see text).

Chr #	Total # Genes	Total # Exons	Total # Introns	Max # Exons/Gene	Chromosome Size (bp)	Avg # of Exons/Gene	Avg Length (bp) ± Std Dev		Total Length (bp)		Shortest (bp)			Longest (bp)			Genes/Millions	Intronless Genes
							Exon	Intron	Exon	Intron	Exon	Intron	Gene	Exon	Intron	Gene		
**1**	3592	31,744	28,152	138	248,956,422	8.8	313 ± 705	5283 ± 16,017	9,934,447	148,725,908	3	1	41	12,573	451,448	1,491,100	14.4	544
**2**	2208	24,805	22,597	363	242,193,529	11.2	299 ± 718	6574 ± 21,058	7,407,514	147,572,357	1	14	49	17,969	866,400	1,900,275	9.1	203
**3**	1916	18,292	16,376	118	198,295,559	9.5	317 ± 759	7669 ± 25,134	5,801,464	125,580,914	3	1	21	24,927	842,378	1,502,150	9.7	181
**4**	1234	10,880	9646	84	190,214,555	8.8	343 ± 740	8687 ± 26,112	3,736,564	83,794,301	8	1	44	9856	912,253	1,474,687	6.5	197
**5**	1496	13,193	11,697	90	181,538,259	8.8	342 ± 778	7678 ± 23,233	4,508,195	89,805,936	6	12	43	22,753	772,519	1,519,058	8.2	205
**6**	1793	16,106	14,313	146	170,805,979	9.0	326 ± 719	6876 ± 20,012	5,256,442	98,421,513	6	1	50	15,177	478,750	1,987,246	10.5	227
**7**	1629	15,280	13,651	108	159,345,973	9.4	315 ± 763	7770 ± 24,375	4,806,589	106,063,166	2	1	53	21,017	657,297	2,304,636	10.2	187
**8**	1207	10,041	8834	86	145,138,636	8.3	335 ± 779	8354 ± 26,109	3,363,013	73,802,469	5	12	23	15,980	955,098	2,059,454	8.3	160
**9**	1402	12,839	11,437	98	138,394,717	9.2	314 ± 714	6073 ± 16,910	4,029,327	69,453,705	3	5	54	10,345	344,501	2,298,478	10.1	200
**10**	1346	12,626	11,280	68	133,797,422	9.4	321 ± 723	7821 ± 23,764	4,058,525	88,222,345	5	67	50	11,090	482,575	1,783,674	10.1	131
**11**	2112	17,709	15,597	90	135,086,622	8.4	311 ± 959	5306 ± 20,073	5,501,031	82,757,607	2	1	50	91,671	811,152	1,468,409	15.6	377
**12**	1773	17,618	15,845	173	133,275,309	9.9	300 ± 686	5076 ± 15,654	5,281,875	80,428,519	9	5	50	14,194	403,400	1,249,864	13.3	169
**13**	715	5982	5267	83	114,364,328	8.4	339 ± 957	9101 ± 29,323	2,025,232	47,933,871	5	66	48	37,567	740,920	1,468,616	6.3	94
**14**	1171	9771	8600	116	107,043,718	8.3	319 ± 734	6212 ± 20,436	3,115,048	53,426,644	4	14	46	17,546	479,079	1,464,560	10.9	229
**15**	1188	11,480	10,292	104	101,991,189	9.7	306 ± 698	5672 ± 17,698	3,514,500	58,372,375	8	21	33	11,532	732,200	887,042	11.6	220
**16**	1468	13,212	11,744	63	90,338,345	9.0	285 ± 607	3892 ± 17,423	3,766,310	45,712,994	3	1	51	10,024	778,855	1,694,208	16.3	159
**17**	2069	19,482	17,413	85	83,257,441	9.4	289 ± 615	3531 ± 13,106	5,622,686	61,484,756	8	1	47	10,345	1,043,910	1,143,719	24.9	251
**18**	495	4568	4073	75	80,373,285	9.2	362 ± 850	10,552 ± 25,422	1,653,586	42,976,591	9	75	50	14,862	411,175	1,195,732	6.2	58
**19**	2449	18,534	16,085	106	58,617,616	7.6	297 ± 655	2382 ± 6158	5,507,290	38,316,474	6	2	47	21,693	255,789	301,152	41.8	358
**20**	983	8035	7052	80	64,444,167	8.2	312 ± 675	5515 ± 18,323	2,509,662	38,891,514	8	31	50	10,441	544,980	2,057,697	15.3	103
**21**	463	3745	3282	47	46,709,983	8.1	309 ± 728	6401 ± 18,061	1,157,367	21,006,837	9	9	60	13,351	323,564	834,698	9.9	95
**22**	797	7029	6232	55	50,818,468	8.8	312 ± 712	4321 ± 12,968	2,194,256	26,927,006	4	1	52	12,955	355,998	701,852	15.7	84
**X**	1152	8062	6910	84	156,040,895	7.0	350 ± 873	6898 ± 24,018	2,820,104	47,662,441	7	67	48	37,027	536,479	1,368,337	7.4	244
**Y**	198	1318	1120	46	57,227,415	6.7	259 ± 540	10,637 ± 33,468	341,789	14,179,080	22	67	64	8690	493,512	686,139	3.5	11

**Table 2:** Exons and introns distribution across human coding genes. Human genome data was downloaded from the UCSC main table browser (GCRh38, December 2013 build). Data was processed using tabular software. Analysis was performed using 26,177 coding genes, containing 278,420 exons and 252,243 introns. See the caution in Table 1 for the shortest introns (<30–40 nt).

Chr #	Total # Genes	Total # Exons	Total # Introns	Max # Exons/Gene	Chromosome Size (bp)	Avg # of Exons/Gene	Avg Length (bp) ± Std Dev		Total Length (bp)		Shortest (bp)			Longest (bp)			Genes/Millions	Intronless Genes
							Exon	Intron	Exon	Intron	Exon	Intron	Gene	Exon	Intron	Gene		
**1**	2729	28,554	25,825	138	248,956,422	10.5	307 ± 702	5136 ± 15,685	8,778,766	132,633,451	3	1	270	12,573	451,448	1,491,100	11.0	149
**2**	1653	22,244	20,591	363	242,193,529	13.5	294 ± 723	6283 ± 20,404	6,534,873	128,437,100	1	37	582	17,969	866,400	1,900,275	6.8	35
**3**	1448	16,336	14,888	118	198,295,559	11.3	312 ± 765	7535 ± 24,995	5,098,251	5,098,251	3	3	294	24,927	24,927	1,502,150	7.3	44
**4**	989	9991	9002	84	190,214,555	10.1	338 ± 729	8471 ± 25,955	3,372,765	76,252,775	8	1	576	9856	912,253	1,474,687	5.2	107
**5**	1103	11,709	10,606	90	181,538,259	10.6	337 ± 785	7240 ± 22,309	3,944,829	76,788,283	6	21	530	22,753	772,519	1,519,058	6.1	85
**6**	1432	14,660	13,228	146	170,805,979	10.2	317 ± 713	6665 ± 19,530	4,653,325	88,160,529	6	1	354	15,177	478,750	1,987,246	8.4	109
**7**	1189	13,187	11,998	108	159,345,973	11.1	306 ± 744	7897 ± 24,804	4,032,145	94,745,609	2	1	600	14,889	657,297	2,304,636	7.5	55
**8**	869	8779	7910	86	145,138,636	10.1	327 ± 768	7942 ± 24,970	2,871,568	62,820,216	5	12	663	15,980	955,098	2,059,454	6.0	26
**9**	1041	11,352	10,311	98	138,394,717	10.9	304 ± 703	5971 ± 16,684	3,453,046	61,566,944	3	5	411	10,345	344,501	2,298,478	7.5	73
**10**	978	11,096	10,118	68	133,797,422	11.3	310 ± 715	7926 ± 24,491	3,440,785	80,199,711	5	67	563	11,090	482,575	1,783,674	7.3	26
**11**	1703	16,196	14,493	90	135,086,622	9.5	301 ± 665	5136 ± 19,917	4,880,180	74,431,941	2	1	484	18,173	811,152	1,468,409	12.6	211
**12**	1407	16,186	14,779	173	133,275,309	11.5	294 ± 688	4939 ± 15,284	4,757,204	72,993,897	9	5	396	14,194	403,400	1,249,864	10.6	50
**13**	426	4920	4494	83	114,364,328	11.5	330 ± 864	8615 ± 29,149	1,624,165	38,717,496	5	66	1310	21,022	740,920	1,468,616	3.7	10
**14**	832	8745	7913	116	107,043,718	10.5	319 ± 741	6107 ± 20,447	2,786,125	48,327,558	4	14	465	17,546	479,079	1,464,560	7.8	38
**15**	769	9686	8917	104	101,991,189	12.6	299 ± 689	5508 ± 16,227	2,900,248	49,116,901	8	21	918	10,227	550,366	887,042	7.5	23
**16**	1117	11,738	10,621	63	90,338,345	10.5	281 ± 607	3831 ± 17,251	3,296,823	40,686,903	5	1	397	10,024	778,855	1,694,208	12.4	20
**17**	1619	17,644	16,025	85	83,257,441	10.9	280 ± 605	3427 ± 13,031	4,943,096	54,914,295	8	1	445	9719	1,043,910	1,143,719	19.4	68
**18**	364	4082	3718	75	80,373,285	11.2	360 ± 872	10,356 ± 24,257	1,469,520	38,504,335	9	75	906	14,862	411,175	1,195,732	4.5	17
**19**	1904	16,757	14,853	106	58,617,616	8.8	296 ± 659	2283 ± 5233	4,964,098	33,906,702	6	2	541	21,693	121,730	301,152	32.5	54
**20**	741	7114	6373	80	64,444,167	9.6	307 ± 669	5564 ± 18,912	2,180,506	35,459,411	11	66	666	10,441	544,980	2,057,697	11.5	23
**21**	311	3183	2872	47	46,709,983	10.2	292 ± 700	5831 ± 16,574	930,502	16,745,676	9	9	147	11,938	323,564	834,698	6.7	54
**22**	598	6190	5592	55	50,818,468	10.4	300 ± 702	4156 ± 11,646	1,857,403	23,242,726	8	1	686	12,955	322,908	701,852	11.8	14
**X**	854	7222	6368	84	156,040,895	8.5	344 ± 774	6428 ± 22,011	2,483,142	40,934,557	10	67	501	10,363	536,479	1,368,337	5.5	79
**Y**	101	849	748	46	57,227,415	8.4	267 ± 576	9483 ± 32,196	226,383	5,888,994	24	67	737	8690	493,512	686,139	1.8	9

**Table 3:** Exons and introns distribution across human non-coding genes. Human genome data was downloaded from the UCSC main table browser (GCRh38, December 2013 build). Data was processed using tabular software. Analysis was performed using 8679 non-coding genes containing 33,931 exons and 25,252 introns. See the caution in Table 1 for the shortest introns (<30–40 nt).

Chr #	Total # Genes	Total # Exons	Total # Introns	Max # Exons/Gene	Chromosome Size (bp)	Avg # of Exons/Gene	Avg length (bp) ± Std Dev		Total length (bp)		Shortest (bp)			Longest (bp)			Genes/Millions	Intronless Genes
							Exon	Intron	Exon	Intron	Exon	Intron	Gene	Exon	Intron	Gene		
**1**	863	3190	2327	46	248,956,422	3.7	362 ± 731	6916 ± 19,245	1,155,681	16,092,457	4	44	41	11,846	300,899	670,478	3.5	395
**2**	555	2561	2006	55	242,193,529	4.6	341 ± 668	9539 ± 26,653	872,641	19,135,257	3	14	49	11,633	415,325	1,126,123	2.3	168
**3**	468	1956	1488	45	198,295,559	4.2	360 ± 702	9009 ± 26,455	703,213	13,405,364	12	67	21	8244	427,004	581,065	2.4	137
**4**	245	889	644	36	190,214,555	3.6	409 ± 847	11,710 ± 28,055	363,799	7,541,526	12	60	44	9848	250,403	491,647	1.3	90
**5**	393	1484	1091	30	181,538,259	3,8	380 ± 722	11,932 ± 30,494	563,366	13,017,653	15	12	43	8875	340,222	932,203	2.2	120
**6**	361	1446	1085	36	170,805,979	4.0	417 ± 772	9457 ± 25,027	603,117	10,260,984	7	73	50	8695	326,934	621,277	2.1	118
**7**	440	2093	1653	48	159,345,973	4.8	370 ± 871	6847 ± 20,981	774,444	11,317,557	13	70	53	21,017	414,132	630,440	2.8	132
**8**	338	1262	924	29	145,138,636	3.7	389 ± 844	11,886 ± 34,163	491,445	10,982,253	15	71	23	12,722	499,303	541,308	2.3	134
**9**	361	1487	1126	45	138,394,717	4.1	388 ± 791	7004 ± 18,841	576,281	7,886,761	14	68	54	7835	308,685	310,090	2.6	127
**10**	368	1530	1162	28	133,797,422	4.2	404 ± 776	6904 ± 16,076	617,740	8,022,634	15	73	50	7617	212,605	337,030	2.8	105
**11**	409	1513	1104	29	135,086,622	3.7	410 ± 2455	7541 ± 21,908	620,851	8,325,666	5	44	50	91,671	295,436	663,821	3.0	166
**12**	366	1432	1066	27	133,275,309	3.9	366 ± 666	6974 ± 19,998	524,671	7,434,622	17	15	50	10,432	266,879	373,979	2.7	119
**13**	289	1062	773	26	114,364,328	3.7	378 ± 1306	11,923 ± 30,179	401,067	9,216,375	14	76	48	37,567	330,963	562,471	2.5	84
**14**	339	1026	687	46	107,043,718	3.0	321 ± 679	7422 ± 20,278	328,923	5,099,086	23	76	46	8430	289,502	437,743	3.2	191
**15**	419	1794	1375	35	101,991,189	4.3	342 ± 742	6731 ± 25,217	614,252	9,255,474	10	21	33	11,532	732,200	797,140	4.1	197
**16**	351	1474	1123	50	90,338,345	4.2	319 ± 601	4476 ± 18,977	469,487	5,026,091	3	47	51	7148	368,335	531,096	3.9	139
**17**	450	1838	1388	61	83,257,441	4.1	370 ± 699	4734 ± 13,885	679,590	6,570,461	15	70	47	10,345	220,687	325,488	5.4	183
**18**	131	486	355	22	80,373,285	3.7	379 ± 642	12,598 ± 35,371	184,066	4,472,256	26	89	50	4791	326,668	545,072	1.6	41
**19**	545	1777	1232	30	58,617,616	3.3	306 ± 611	3579 ± 12,785	543,192	4,409,772	11	62	47	11,194	255,789	292,306	9.3	304
**20**	242	921	679	43	64,444,167	3.8	357 ± 720	5055 ± 11,393	329,156	3,432,103	8	31	50	10,441	138,007	195,695	3.8	80
**21**	152	562	410	32	46,709,983	3.7	404 ± 869	10,393 ± 25,888	226,865	4,261,161	16	79	60	13,351	256,374	539,254	3.3	41
**22**	199	839	640	30	50,818,468	4.2	401 ± 775	5757 ± 21,234	336,853	3,684,280	4	9	52	8320	355,998	411,958	3.9	70
**X**	298	840	542	17	156,040,895	2.8	401 ± 1472	12,413 ± 40,403	336,962	6,727,884	7	78	48	37,027	405,107	1,033,350	1.9	165
**Y**	97	469	372	26	57,227,415	4.8	246 ± 467	16,221 ± 58,381	115,406	1,313,894	22	90	64	5836	353,508	320,464	1.7	2

In contrast to abundant intronless genes, coding and non-coding genes rarely contain more than 100 or 50 exons respectively (15 coding and 7 non-coding genes). The absolute record is held by the titin gene, which contains 363 exons (*TTN*; chr2:178,525,990–178,807,423). However, overall, a typical coding gene contains 10.8 exons and 9.8 introns (Table 2). In contrast, non-coding genes appear to contain 3.9 exons and 2.9 introns on average (Table 3).

## 3. Introns May Contain Independent Coding and Non-Coding Genes

Gene distribution in the human genome is not uniform, some regions being free of genes and others where genes are closely packed. In some cases nearby genes can even overlap. A peculiar case of overlapping genes are nested genes, *i.e.*, independent transcription units entirely included within the bounds of an intron, or more rarely of an exon, of a larger gene (reviewed in [12]). About 10% of *Drosophila melanogaster* genes [13], but only ~1% (373) of human genes [14], were assigned to nested positions, two-thirds of them being transcribed from the strand opposite to that of their host gene. About 58% of the nested/host pairs were conserved in the mouse and some were even present in chicken and fish, while nested pseudogenes are only poorly conserved [14]. The majority of these nested genes code for proteins that are functionally unrelated to that encoded by their host genes [14].

**Figure 2:**
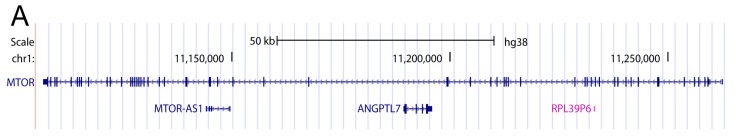

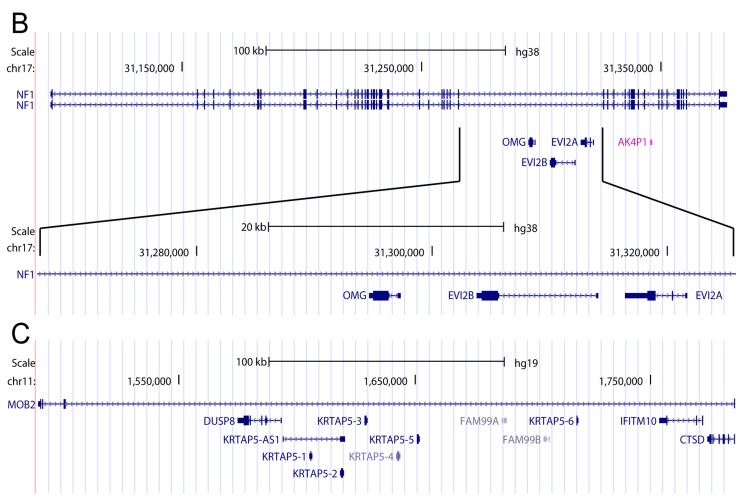
Selected tracks for the human *MTOR* (**A**); *NF1* (**B**) and *MOB2* (**C**) genes. (**A**) Mechanistic target of rapamycin (serine/threonine kinase) gene (*MTOR*; chr1:11,166,588–11,322,608) is composed of 58 exons spanning 156,020 nucleotides. It contains 3 nested genes: *MTOR-AS1* (MTOR antisense RNA 1; chr1:11,203,955–11,209,595) gene which encodes an antisense RNA across introns 24 to 27 and overlapping exon 25; *ANGPTL7* (chr1:11,249,346–11,256,038) gene embedded in intron 30; *RPL39P6* (chr1:11,293,020–11,293,169) gene which is a pseudogene included in intron 43; (**B**) Neurofibromin 1 gene (*NF1*; chr17:29,421,945–29,704,695) composed of 58 exons spanning 282,751 nucleotides holds 4 nested genes, 3 of which are coding genes located in intron 30 and 1 is a pseudogene (*AK4P1*; chr17:29,672,539–29,673,205; included in intron 42), all of which are transcribed from the opposite strand. The 3 nested genes are the oligodendrocyte myelin glycoprotein gene (*OMG*; chr17:29,621,668–29,624,380), ecotropic viral integration site 2A (*EVI2A*; chr17:29,643,428-29,648,767) and 2B (*EVI2B*; chr17:29,630,788–29,641,130); (**C**) MOB kinase activator 2 gene (*MOB2*; chr11:1,490,685–1,785,501) contains 13 nested genes: dual specificity phosphatase 8 (*DUSP8*) coding gene; KRTAP5-1/KRTAP5-2 antisense RNA 1 (*KRTAP5-AS1*) pseudogene; keratin associated protein 5-1 (*KRTAP5-1*) and 5 other paralog genes (*KRTAP5-2* to *-6*); family with sequence similarity 99, member A (*FAM99A*) and member B (*FAM99B*) which are non-coding genes; interferon induced transmembrane protein 10 (*IFITM10*) and cathepsin D (*CTSD*) coding genes; one unannotated gene corresponding to EST GenBank AF085962. Amazingly, *KRTAP5-1* gene was embedded within and oriented in the opposite direction of *KRTAP5-AS1* pseudogene intron, itself oriented in the opposite direction to MOB2 intron.

Nested genes that produce small regulatory RNAs independently of the transcription of the host gene have also been described [12,14]. Examples of coding and non-coding nested genes are illustrated in Figure 2. With respect to miRNA genes, independent transcription units account for about 350 miRNA genes (about half of all intronic miRNAs which themselves account for about half of total miRNAs in the human genome; miRBase release 17.0). These intronic miRNAs under the control of their own promoter have to be distinguished from other intronic miRNAs whose production is strictly dependent on transcription of the host gene (see Section 6. below) and from so-called mirtrons or most of the snoRNAs that rely on splicing of the host mRNA to be produced (see below) [15,16,17]. Intriguingly, at least one miRNA, miRNA-128-2, can be expressed from two different promoters and by two different polymerases (Pol II and Pol III) [18,19]. Among the other small ncRNAs, transfert RNAs (tRNAs) can also be hosted in other genes. In the human genome, out of the 497 tRNA genes and 324 tRNA-derived pseudogenes supposed to be no longer functional, 47 (about 10%) were located in intron of coding genes and 32 (about 10%) in intron of non-coding genes (hg19). For example, the tRNA-Leu (anticodon TAA; chr6:69,204,486–69,204,568) is located in the intron 17 of the human brain-specific angiogenesis inhibitor 3 (*BAI3*; chr6:69,232,406–69,389,511) gene.

Although it has been proposed that a compact genome allows for quicker replication rate in prokaryotes, the functional benefits for mammalian genomes to hold nested genes are still unclear. Still, the presence of nested transcription units in introns raises the questions of their concerted transcription and regulation. It is likely that the host gene and its nested gene transcribed from the opposite strand are inversely regulated and transcribed, owing to promoter competition or steric hindrance of RNA polymerase and transcription factors. In contrast, if the nested and host genes are transcribed from the same strand, it is quite straightforward to predict a concerted model of transcriptional regulation of both genes to allow expression, if not in the same pathways, at least in the same cellular context or in response to the same environmental cues.

## 4. When Introns Are too Big to Be Spliced at Once—Intron Re-Splicing

**Figure 3:**
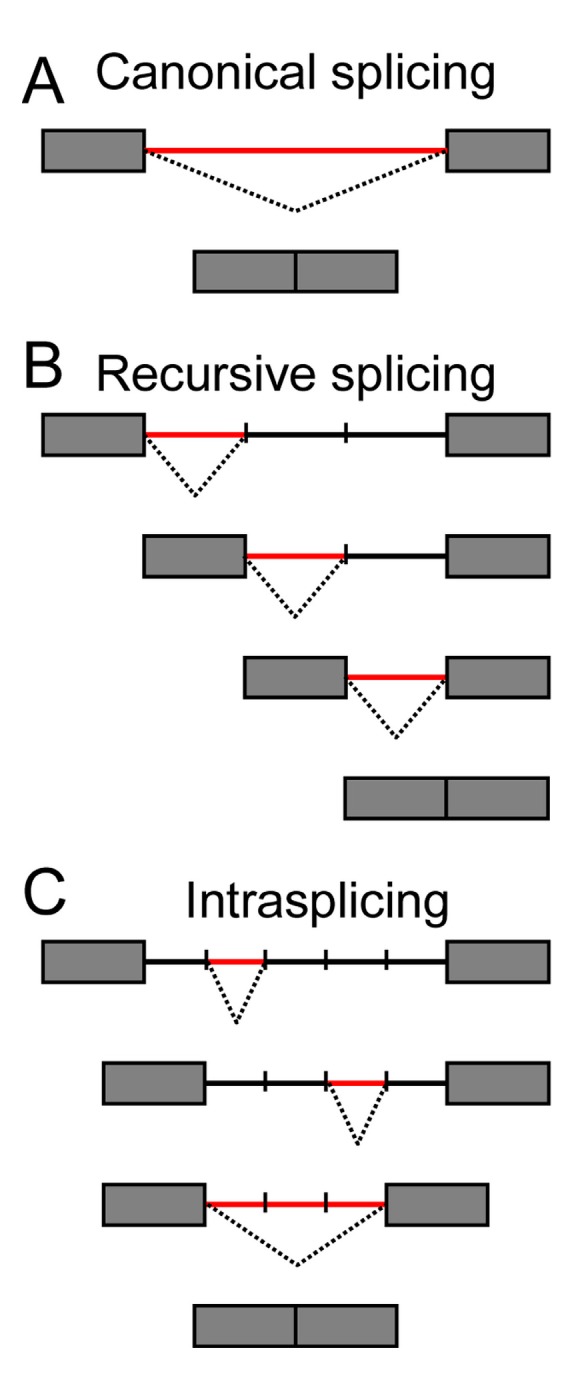
Schematic representation of canonical- (**A**), recursive- (**B**), and intra- (**C**) splicing. Boxes and lines are exons and introns, respectively. Splicing processes are shown by dotted lines.

Through classical mechanisms than we will not detail here, pre-mRNA splicing is precisely controlled in a spatial and temporal manner to generate mature RNAs (For a review, see [20]). If one can easily understand the mechanisms by which “normal” introns (100–1000 nucleotides) are excised, it is more difficult to visualize the case of long or very long introns, in particular when it involves the formation of a lariat as this is the case for spliceosomal introns. In fact, a multi-step process called re-splicing has been proposed for some of these huge introns, along which long introns are removed in successive reactions. Besides canonical splicing (Figure 3A), at least two additional pathways have been described to date (Figure 3B,C):

(1) The recursive splicing consists in the stepwise removal of introns by sequential splicing reactions starting from the most 5' donor site and progressing towards the most 3' acceptor site [21,22]. To date, recursive splicing has not been found in vertebrate cells. The most studied recursive splicing process is that of the ultrabithorax (Ubx) gene in the fruit fly.

(2) The intra-splicing or nested splicing first requires internal splicing, using internal canonical donor and acceptor sites, followed by external splicing involving the most outer donor and acceptor sites [23]. This process thus involves multiple splicing events within a large intron, to shorten it and bring it to a size that can be handled by conventional splicing machineries and mechanisms. To our knowledge, these splicing events, despite the fact that each of them represents an intermediate step in the entire process, do not have particular characteristics that make them distinguishable from classical splicing mechanisms. Ribonucleoprotein complexes seem to be identical between nested and canonical splicing and they both involve the formation of intermediate lariat structures.

The human dystrophin gene (*DMD*; chrX:31,137,345–33,357,726) is one of the largest annotated genes, spanning more than 2 Mbp and generating transcripts containing up to 79 coding exons. Strikingly, *DMD* transcripts are only 14 kb long since more than 99% of the gene sequence is composed of introns, whose lengths vary from 107 nt (intron 14) to 248,401 nt (intron 44) [23]. Intron 7 (chrX:32,717,411–32,827,609) of the Dp427c isoform (NM_000109) is spliced out through an intrasplicing mechanism [24]. Another example is the erythrocyte Membrane Protein Band 4.1 (*EPB41*) gene in which Exon 1A and exon 2 are joined through two nested splicing events [25].

## 5. Cytoplasmic Splicing—Adding to the Complexity of Transcriptional Regulation

In eukaryotes, splicing occurs in the nucleus in a transcription-coupled manner where the spliceosome complex catalyzes intron removal, deposits exon–junction complexes (EJC) to mark sites of intron removal but also participates in nuclear retention of incompletely spliced pre-mRNAs. Quality control of incompletely spliced pre-mRNAs to prevent their translation into aberrant proteins involves at least two mechanisms; the nuclear degradation of pre-mRNAs that failed to be exported to the cytoplasm, and the cytoplasmic degradation through nonsense-mediated decay (NMD) of intron-retaining RNAs with a premature stop codon 5' of an EJC [26].

The major spliceosome comprises 5 small nuclear RNAs (snRNA) and a multitude of associated protein factors to form small nuclear ribonucleoprotein particles (snRNPs), U1, U2, U4, U5, and U6 [27]. Spliceosome assembly occurs on pre-mRNAs through consensus sequences described in the preamble [2].

A minor spliceosome has been reported in plants, insects, and vertebrates, which is specialized in the splicing of a rare class of introns (less than 0.5% of all introns, which is still about 5000 introns as referred to Table 2) with unusual donor and branch signals and atypical and diverse terminal dinucleotides [28,29]. These introns are denoted U12-type introns as opposed to classical U2-type introns, as they use base-pairing of U12 at the branch point sequence instead of U2. Indeed, minor spliceosome uses an assembly of less abundant snRNPs, namely U11, U12, U4atac, and U6atac [28,29].

Thus, although they represent a minority of introns, highly evolutionary conserved U12-type introns are associated with a sophisticated parallel splicing system that is essential for cell division of vertebrates [30]. In addition to holding distinct functions, the possibility that this minor splicing pathway is spatially separated in the cell from the major pathway may well represent a mean through which eukaryotic cells extended their regulatory repertoire [30]. Indeed, there is still a debate about where the minor spliceosome operates, either in the cytoplasm [30] or in the nucleus [31], although evidence for the former accumulates:

(1) Transcripts with unspliced minor introns can be found in the cytoplasm, together with components of the minor spliceosome. In addition, the specific inhibition of minor spliceosome components in the cytoplasm, using antisense morpholino conjugated to a nuclear-export sequence, leads to accumulation of unspliced U12-introns in the cytoplasm [30].

(2) The minor spliceosome, unlike its major counterpart, seems to evade mitotic down-regulation, to be able to operate during mitosis and to function independently of transcription [30,32,33]. In a context when transcription and major splicing are down-regulated, while nuclear envelope breakdown releases potentially partially unspliced transcripts into the cytoplasm of the dividing cell, minor splicing could be available to control the fate of certain transcripts. The presence of a minor spliceosome is actually a distinguishing feature of species with open mitosis.

(3) Along the same lines, platelets, which are specialized hemostatic cells that circulate in the blood as anucleate cytoplasts, contain essential spliceosomal proteins and small nuclear ribonucleic acids (snRNAs), as well as a subset of pre-mRNAs that are further spliced into mature message in response to platelet activation [34]. This mechanism would allow platelets to respond to external signals triggered by vascular wall injury even in a context that is devoid of direct nuclear regulation.

(4) Additional support for the functionality of RNA splicing in the cytoplasm was provided in neurons. Neurons have a cell body containing the nucleus and core cytoplasmic components surrounded by highly specialized cytoplasmic extensions called dendrites and axons. Certain not fully spliced intron-retaining mRNAs are directed toward neuronal dendrites where they fully mature [35,36]. As mRNA can make hundreds of proteins on site, it is assumed that localized mRNA avoids the significant energy costs of moving protein molecules individually. Similarly, localized splicing like in dendrites of neurons, and presumably in other cell types, may serve as a regulatory switch to produce translatable mRNAs when and where the protein is needed.

(5) Cytoplasmic splicing is one of the major regulatory mechanisms of the unfolded protein response (UPR), although it occurs through unconventional mechanisms [37]. Splicing of X-box binding protein 1 (XBP1) and Homologous to Atf/Creb1 (HAC1) pre-mRNAs, regulators of the UPR, occurs in the cytoplasm using a kinase with endoribonuclease activity (IRE1) followed by joining of exons by an RNA ligase [37,38,39,40]. HAC1 mRNA is exported to the cytoplasm as an intron-retaining mRNA preventing complete translation of polyribosome-associated HAC1 mRNAs [37,38,39,40]. This mechanism provides a way to attenuate translation of the transcription factor Hac1p in cells in which the UPR is not induced.

In sum, cytoplasmic splicing offers an efficient way to diversify cellular phenotypes without the need for nuclear activation of sets of genes as well as allowing for rapid regulation of local environment and subcellular proteomes and hence, for a quasi instant response to environmental cues.

## 6. Splicing of Introns to Produce Small Non-Coding Transcripts

miRNAs play pivotal roles in diverse biological functions, mainly through the repression of target genes. Less than half of intronic miRNAs are transcribed independently of their host gene, as long primary capped and polyadenylated transcripts (pri-miRNAs) by RNA polymerase II (RNA pol II), which undergo a series of maturation steps via the formation of a hairpin-forming precursor (pre-miRNA) until the production of mature miRNAs in the cytoplasm. These intronic miRNAs should be named intragenic or nested miRNAs to distinguish them from independent intergenic miRNAs. In contrast, the other half of so-called intronic miRNAs share promoter and regulatory elements with their host gene [18,19,41,42,43]. As a consequence, the pre-mRNA of the host gene also serves as the pri-miRNA that is processed as described above [44,45,46]. Some examples of intronic snoRNAs and miRNAs along with their host genes are described in Table 4.

Interestingly, there are cases where small regulatory RNAs are directly produced by way of splicing of the intron from the host transcript and thus, are strictly dependent on both transcription and splicing of the host gene.

In the case of snoRNAs, while some have their own promoter and are transcribed by RNA pol II, the majority is produced through splicing of the host intron. Whereas most of the host transcripts are mRNAs, some examples of long non-coding RNA (lncRNA) hosting snoRNAs exist such as the growth arrest-specific transcript 5 (GAS5) that contains 10 different snoRNAs, almost one in each one of its introns (all but the intron 9/11) [47]. Noteworthy, there is a class of genes called Small Nucleolar RNA Host Gene (SNHG) whose mRNAs produced after the maturation steps are degraded, the only stable isoforms generated by the splicing process being those retaining snoRNA-hosting introns [47]. There are 19 annotated SNHG genes in humans hosting 53 snoRNAs (from 1 to 15 snoRNA per gene; *GRCh38*).

**Figure 4:**
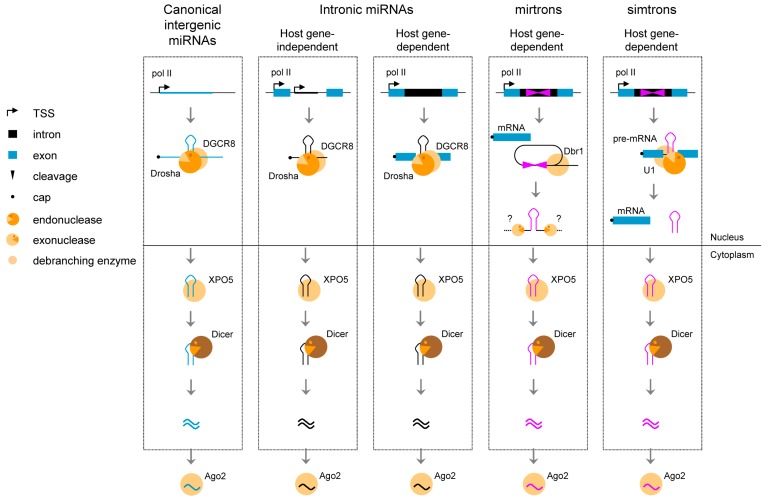
Different mechanisms of microRNA biogenesis. The first three panels correspond to the canonical miRNA pathway, either intergenic (miRNAs) or intronic (intronic miRNAs), and the last two panels represent new alternative pathways, either independent of the microprocessor Drosha/DGCR8 (mirtrons) or independent of DGCR8 but dependent of U1 snRNP (simtrons), both dependent on splicing to produce miRNAs. DGCR8 stands for DiGeorge syndrome critical region gene 8. Adapted from [48].

At least two alternative pathways in the biogenesis of miRNAs use splicing to produce pre-miRNA-like transcripts independently of the microprocessor complex: the mirtron and the simtron pathways (Figure 4).

**Table 4:** Some examples of intronic snoRNAs and miRNAs and their host genes.

Name		Genome Position	Host Intron	Host Gene	Genome Position	Gene Function
**snoRNA**	*ACA67*	chr21:33,749,496–33,749,631	Intron 5	URB Ribosome Biogenesis 1 homolog (URB1)	chr21:33,683,330–33,765,312	Ribosome biogenesis
	*HBI-43*	chr20:17,943,353–17,943,589	Intron 1	Sorting Nexin 5 (SNX5)	chr20:17,922,244–17,949,490	Member of the sorting nexin family, involved in intracellular trafficking
	*SNORD119*	chr20:2,443,605–2,443,686	Intron 2	Small Nuclear Ribonucleoprotein Polypeptides B and B1 (SNRPB)	chr20:2,442,288–2,451,499	Nuclear proteins that are found in common among U1, U2, U4/U6, and U5 small ribonucleoprotein particles (snRNPs)
	*U101*	chr6:133136446–133136518	Intron 3	Ribosomal protein S12 (RPS12)	chr6:133,135,708–133,138,703	Component of the ribosomal 40S subunit
	*HBII-429*	chr6:133137941–133138016	Intron 4	Ribosomal protein S12 (RPS12)	chr6:133,135,708–133,138,703	Component of the ribosomal 40S subunit
	*ACA33*	chr6:133138358–133138490	Intron 5	Ribosomal protein S12 (RPS12)	chr6:133,135,708–133,138,703	Component of the ribosomal 40S subunit
	*ACA37*	chr18:51,748,654–51,748,782	Intron 1	Methyl-CpG Binding Domain protein 2 (MBD2)	chr18:51,677,971–51,751,158	Repress transcription from methylated gene promoters
**miRNA**	*hsa-mir-643*	chr19:52,785,050–52,785,146	Intron 1	Zinc Finger protein 766 (ZNF766)	chr19:52,772,824–52,795,976	Unknown
	*hsa-mir-220c*	chr19:49,063,529–49,063,611	Intron 1	Sulfotransferase family, cytosolic, 2B, member 1 (SULT2B1)	chr19:49,055,429–49,102,684	Catalyze the sulfate conjugation of many hormones, neurotransmitters, drugs, and xenobiotic compounds
	*hsa-mir-3191*	chr19:47,730,201–47,730,276	Intron 2	BCL2 Binding Component 3 (BBC3)	chr19:47,724,079–47,736,023	Member of the BCL-2 family of proteins, cooperates with direct activator proteins to induce mitochondrial outer membrane permeabilization and apoptosis
	*hsa-mir-770*	chr14:101,318,727–101,318,824	Intron 9	Maternally Expressed 3 (non-protein coding) (MEG3)	chr14:101,292,445–-101,327,360	Long ncRNA tumor suppressor. Interacts with the tumor suppressor p53, and regulates p53 target gene expression
	*hsa-mir-1273d*	chr1:10287776–10287861	Intron 1	Kinesin family member 1B (KIF1B)	chr1:10,270,764–10,441,661	Transports mitochondria and synaptic vesicle precursors
	*hsa-mir-3190*	chr19:47,730,199–47,730,278	Intron 2	BCL2 Binding Component 3 (BBC3)	chr19:47,724,079–47,736,023	Member of the BCL-2 family of proteins, cooperates with direct activator proteins to induce mitochondrial outer membrane permeabilization and apoptosis
	*hsa-mir-942*	chr1:117,637,265–117,637,350	Intron 18	Transcription Termination Factor, RNA polymerase II (TTF2)	chr1:117,602,949–117,645,491	Member of the SWI2/SNF2 family of proteins

Unlike canonical miRNAs that require the microprocessor complex to produce the hairpin pre-miRNA, the so-called mirtron pathway is thought to require debranching of the lariat-intron produced by splicing, by a debranching enzyme dbr1, as a prerequisite to produce a hairpin pre-miRNA-like structure [49]. Until now, hundreds of mirtrons have been predicted through bioinformatical approaches although the mirtron origin, *i.e.*, dependency on splicing and debranching machineries, has been experimentally validated for a handful of them [50]. More recently, another alternative pathway to generate miRNAs (simtrons) from introns has been described, which also relies on splicing factors such as U1 snRNP and on the microprocessor enzyme Drosha [51,52] but not on the debranching enzyme.

Embedding of genetic information for small regulatory RNAs within a longer mRNA or ncRNA provides an attractive concerted model for eukaryotic gene regulation, whereby multiple actors in the same pathways or in a given cellular context are produced from interdependent transcripts. The co-linear expression of miRNA and protein-coding genes may also function to ensure negative feedback on the protein-coding gene to prevent its over-expression. Indeed, while some intronic miRNAs are transcribed from their own promoter as independent transcription units, expression of intronic miRNAs has been widely linked to the regulation of expression of their host gene [43,53]. Furthermore, approximately 20% of intronic miRNAs (56 out of 296) have been predicted to target their own host transcript, either through recognition of the 3'-UTR, or indirectly through the transcriptional inhibition of a positive regulator of the host gene [54,55]. While there is growing evidence for a complex crosstalk between transcription, splicing and pre-miRNA processing and for a potential co-regulation of miRNA and host gene expression, this relationship is not yet completely understood [44,56,57].

*Splicing of introns may also lead to the production of long non-coding RNAs with a circular structure (ciRNA). In human cells, Zhang and colleagues [58] have recently identified hundreds of such RNAs, ranging from 200 nt to over 3000 nt in length, with no ORF, and suggested to be expressed in a cell-specific manner. The mechanism of biogenesis was described as follows [58]; the lariat structure of introns that contain a consensus 7 nt GU-rich pattern in 5' splice site and an 11 nt C-rich element near the branchpoint are digested from the 3' end to the branchpoint to preserve the loop portion of the lariat intron. In contrast to circular RNAs originating from circularized exons and acting as miRNA sponges in the cytoplasm [59,60], intronic ciRNAs seem to function as positive regulators of Pol II transcription and play a role in the efficient transcription of their host gene*.

## 7. Introns Can Be Retained within Coding Segments—When Alternative Splicing of Introns Participates in Proteome Diversification

**Figure 5:**
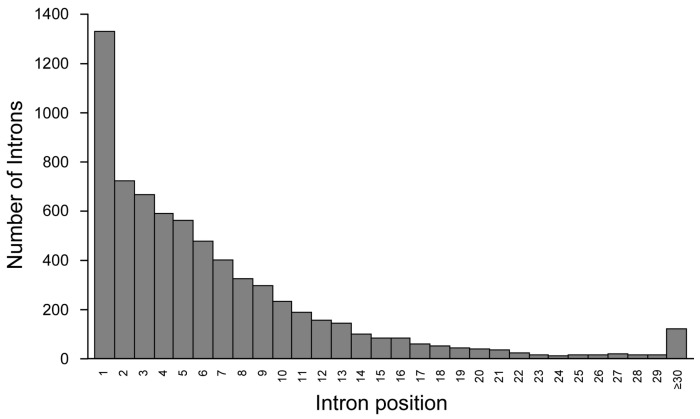
Intron distribution relative to their position in genes. About 20% of all introns subjected to alternative splicing (AS) are intron 1.

Alternative splicing (AS) is a mechanism that allows the inclusion of non-coding sequences (introns) or excision of coding segments (exons) within mature mRNAs. While AS of introns represents a major event in plants, it accounts for only 2 to 5% of all alternative events in humans [61,62]. However, given the 252,243 introns in the human genome (Table 2), this could still contribute to the generation of additional 5044 to 12,612 potential protein isoforms. Actually, 6157 introns from the UCSC Table Brower (Alt Events—Alternative Splicing, Alternative Promoter and Similar Events in UCSC Genes) have been described to be conserved between human and mouse, and further confirmed to exist in EST or cDNA databases, indicating that they may well be included in mRNAs under certain physiological conditions [63]. As shown in Figure 5, introns subjected to AS are located predominantly in 5' of the genes (more than 20% are intron 1).

We already mentioned examples where incompletely spliced intron-retaining transcripts are exported to the cytoplasm where their maturation is completed under certain physiological or cellular contexts. This may seem surprising since, in the majority of cases, intron retention leads to premature stop codons that trigger the cellular surveillance machinery and degradation of such transcripts by the nonsense-mediated decay pathway (NMD) [64]. Why and how some intron-retaining transcripts escape NMD has not been completely solved, but can include the presence of retrotransposon-containing sequences like intron-retaining transcripts in dendritic cells [65].

Besides the already mentioned example of intron-retention that allows for fine tuning of protein production at the right place and at the right moment in dendritic cells and neurons [35,36], intron retention also contributes to the control of tissue specific gene expression. For example, *LY6G5B* and *LY6G6D* genes located in the major histocompatibility complex (MHC) class III region on chromosome 6 can produce leukocyte antigen-6 (Ly6)-producing mRNA as well as intron-retaining non-coding transcripts that seem to be more abundant and stable than the correctly spliced isoform in most cell lines tested, and to escape NMD [66]. This mechanism was proposed to prevent production of LY6 antigens in non-affiliated tissues.

When it is tightly coupled to NMD, orchestrated intron retention allows for fine control of expression levels of proteins involved in the immune response [67]. It also allows for fine control of transcriptional output, through selective degradation via the NMD when intron retention leads to a premature stop codon or through nuclear retention that prevent their translation [68]. Other examples include transcriptional diversification through IR discussed in Section 8.

**Figure 6:**
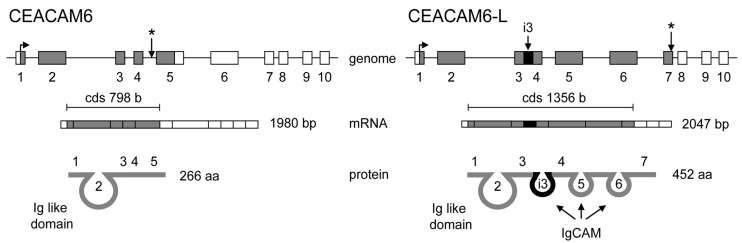
Carcino-embryonic antigen related cell adhesion molecule 6 (CAECAM6) and its novel spliced variant CAECAM6-Long (CAECAM6-L) from rat testis. Gene, mRNA and protein representation reproduced using data from [69]. Exons are numbered. Twisted arrow and star corresponded to ATG and stop codon, respectively. Intron 3 is denoted i3. Colored boxes corresponded to exons, thin line to introns except for i3, which was shown as a black box. White and grey boxes are for untranslated region (UTR) and coding sequences, respectively.

More directly, intron-retention participates in the diversification of the proteome, although, as we already mentioned, it is less commonly used in mammals than in plants. Figure 6 illustrates an attractive case of intron retention taking as an example carcino-embryonic antigen-related cell adhesion molecule 6 (CAECAM6) and a novel spliced variant CAECAM6-Long (CAECAM6-L) specifically expressed in rat testis, which both belong to an immunoglobulin (Ig) superfamily of proteins. The retention of intron 3 in the mature transcript not only adds an IgCAM (immunoglobulin superfamily cell adhesion molecule) domain, but also triggers a shift in the open reading frame leading to the subsequent choice of a more downstream stop codon. This shift adds the end of exons 5 to 7 to coding sequences, which in turn add two additional IgCAM domains. Therefore, Ceacam6 protein contains only one Ig-like domain without the transmembrane region whereas Ceacam6-L has three IgCAM domains and a transmembrane region. Thus, intron retention may add directly (exon 3) or indirectly (exons 5 to 7) three new protein domains conferring novel functions to an adhesion molecule in male germ cells [69].

Other examples of intron-retained transcript together with their biological function in mammals can be found in Table 1 of Buckley *et al.* [70].

## 8. Introns as a Switch to Produce Coding or Non-Coding RNAs—When Alternative Splicing of Introns Generates Transcriptome Diversity

It is now evident that the transcriptional output of mammalian genomes is much more complex than estimates based on the number of protein-coding genes, and that non-coding RNA widely increase the source of regulatory molecules. We recently characterized a new class of ncRNAs, called bifunctional RNAs, *i.e.*, RNAs for which both coding capacity and activity as functional RNA have been described [71,72,73,74,75,76,77,78,79].

Historically, the pioneer member of this new class of RNAs is SRA (Steroid Receptor RNA Activator). SRA was first identified as a structural ncRNA molecule in hormone receptor complexes, characterized by discrete stem-loop structures required for its function as a co-activator [80,81] as extensively reviewed by Cooper *et al.* [72]. A few years later, we identified new SRA isoforms, exhibiting an additional exon upstream of the core exons, containing two initiating methionines and a predicted open reading frame (ORF) of 236/224 amino acids, for which two associated SRA proteins (SRAP) were detected shortly afterwards [71,76,77,82,83]. Interestingly, the existence of both coding and non-coding SRA transcripts seems to be regulated, at least in part, by the differential splicing of the first intron of SRA [76,77]. Whereas all these isoforms exist in the cell, we reported that the balance between coding and non-coding isoforms influences the fate of human muscle progenitors towards self-renewal or differentiation [77,79] which is in total agreement with AS working as a developmental switch.

**Figure 7:**
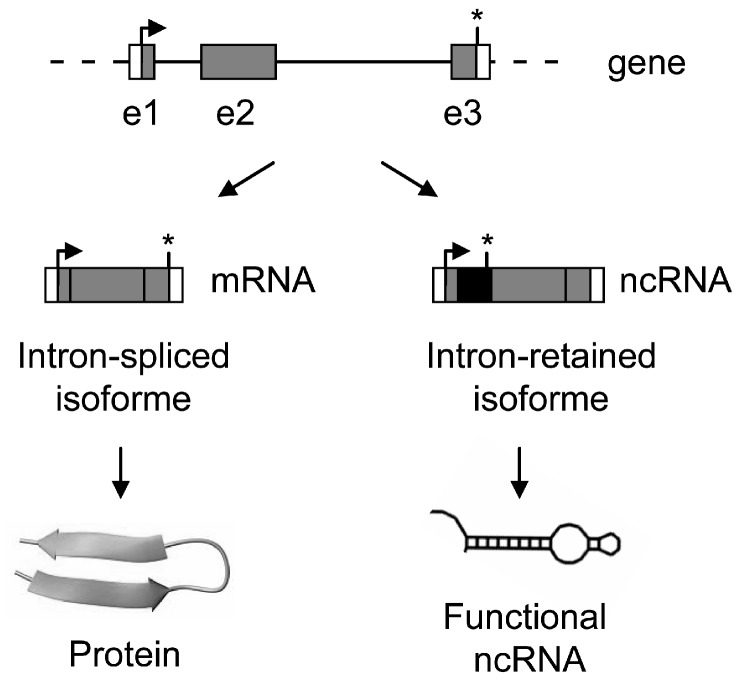
Both coding and non-coding RNAs can be produced by a given genetic locus. Intron-spliced isoforms are translated into protein while intron-retaining transcripts escape surveillance machineries and produce functional ncRNAs. Exons are numbered and represented by grey boxes and by thin lines. Arrow and star represent ATG and stop codon, respectively. In the mature transcript, the retained intron appears as a black box, UTR and coding sequences as white and grey boxes respectively.

An example of such a genetic locus producing both coding and non-coding RNA, depending primarily on an event of AS of intron (Figure 7), is no longer a weird isolated case. First, we recently hypothesized that similar cases, not yet formally tested experimentally, might exist and revealed that the human genome contains around 300 possibilities of potentially new bifunctional RNA, *i.e.*, in which intron retention disrupts the ORF permitting transcription of a ncRNA [78]. Specific examples can also be found in the literature, like the case of Apolipoprotein E (ApoE), a multifunctional protein with three common isoforms (ApoE2, ApoE3, and ApoE4) that play different roles in lipid metabolism and neurobiology. An additional isoform with intron 3 retention (ApoE-I3) has been described [84]. Whereas ApoE-I3 escapes degradation and is stably expressed as a nuclear-retained and non-translated transcript under normal conditions in neurons, a switch towards splicing of intron 3 and expression of the ApoE mRNA that is correctly exported to the cytoplasm and translated into a protein product occurs in response to neuronal injury [84]. More intriguingly, a splicing RNA isoform of the murine liver X receptor (LXR)-β with retained intron 2 has been recently proposed to function as a co-activator of its LXR-β protein counterpart [85]. Conversely, we found that SRA protein SRAP could act as an antagonist of the co-activation function of the non-coding SRA RNA on MyoD-mediated muscle differentiation and forced reprogramming [77,79]. Other examples exist throughout species, which have at least in common that intron-retaining transcripts escape surveillance machineries and that both coding and non-coding RNAs produced by a given genetic locus operate in the same pathways (reviewed in [79]).

Combined, the recent findings that alternative splicing of introns could produce RNAs with coding capacity or acting as functional ncRNAs and that splicing could directly generate small regulatory RNAs raise the fascinating assumption that a single transcription unit could generate multiple molecules including proteins, long or smaller regulatory ncRNAs such as miRNAs, and bring to light the central role of mammalian introns in the diversification of both proteome and transcriptome depending upon the need of the cell to respond to particular environmental settings.

## 9. Concluding Remarks

Introns have been regarded for a long time as “junk DNA” and remnants of archaic ancestral genomes. Questions about their origin and function arose immediately after their discovery in 1977, in particular regarding the energy waste that this represents for the cell to transcribe introns if they have to be eliminated from transcripts. Since then, accumulating evidence lent support to their fundamental importance in the regulation of mammalian gene expression programs, from transcriptional initiation, termination and stability, through recruitment of the exon-junction complex to recruitment of chromatin remodelers through the spliceosome.

With introns representing more than half of the genome in higher eukaryotes and being absent from prokaryotic genomes, the apparent correlation between the genomic fraction occupied by introns and organism complexity must have some functional significance.

In essence, eukaryotic cells use a variety of strategies to control their transcriptional output that employ a large number of regulatory factors that, in turn, must be tightly regulated. We provided examples whereby introns, as genetic entities or RNA segments, facilitate or participate in this amazing regulation feat by sheltering information for small regulatory RNAs allowing for concerted expression of multiple molecules in a given context, influencing where and when a messenger RNA is spliced and translated, preventing or attenuating translation off context or, on the contrary, diversifying the type and function of the molecules produced depending on the internal and external environment.

All these intron-linked mechanisms add some levels of sophistication in the way mammalian cells gain their phenotypic variability and opened a whole new field of investigation where introns take central stage and emerge as key elements in shaping cell identity.
